# Dynamic Boundary Estimation of Suspended Sediment Plume Benefit by the Autonomous Underwater Vehicle Sensing

**DOI:** 10.3390/s24248182

**Published:** 2024-12-21

**Authors:** Yanxin Zhang, Shaoyuan Li

**Affiliations:** Key Laboratory of System Control and Information Processing, Department of Automation, Shanghai Jiao Tong University, Shanghai 200240, China; zhangyanxin1996@sjtu.edu.cn

**Keywords:** dynamic boundary estimation, suspended sediment plume, autonomous underwater vehicle, deep-sea mining, underwater sensing

## Abstract

The suspended sediment plume generated in the deep-sea mining process significantly impacts the marine environment and seabed ecosystem. Accurate boundary estimation can effectively monitor the scope of environmental impact, guiding mining operations to prevent ecological damage. In this paper, we propose a dynamic boundary estimation approach for the suspended sediment plume, leveraging the sensing capability of the Autonomous Underwater Vehicles (AUVs). Based on the plume model and the point-by-point sensor measurements, a Luenberger-type observer is established for designing the AUV control algorithm. To address the challenge of unknown and time-varying environmental parameters, the estimation errors are reduced by using the projection modification unit. Rigorous convergence and stability analyses of the proposed control algorithm are provided by the Lyapunov method. Numerical simulations demonstrate that the improved algorithm enhances the estimation accuracy of unknown parameters and enables the AUV to patrol along the dynamic boundary in a shorter time, thereby verifying the effectiveness of the boundary estimation algorithm based on AUV sensing.

## 1. Introduction

The deep sea is abundant in mineral resources, which has the potential to satisfy the increasing demand for minerals in the future. As mining operations expand, the current challenge is to minimize the impact on marine ecology during the extraction of deep-sea minerals. Taking into account the environmental implications, the primary problems are the removal of bottom materials and the formation of sediment plumes. Specifically, the dispersion of mining tailing plumes can significantly change the marine habitats. Therefore, the monitoring of environmental impacts should focus on the dispersion of tailing plumes from mining vehicles.

Currently, the research on the dispersion of plumes from deep-sea mining is mainly concerned with numerical simulation methods to predict dispersion phenomena. On the one hand, scholars study the plume behaviors under different conditions in laboratory experiments and investigate the key parameters of the model from a theoretical perspective [1]. On the other hand, scholars carry out seafloor disturbance experiments for observing and measuring the plume dispersion, thereby verifying and modifying the model [2,3,4]. However, due to the limitations of real-time computing, complex numerical oceanographic models will take a long time to obtain the outcome, which makes it difficult to timely capture the dynamic boundary.

The objective of tracking the suspended sediment plume is to monitor the contaminant boundary or contours at specific concentrations, which helps determine the extent of pollution and the rate of its propagation. In the early stages, pollution monitoring mainly relied on the real-time information gathered from pre-deployed static sensor networks. However, once these networks were installed, they could not be dynamically adjusted, and they required a specific sensor placement density [5]. Low-density permanent in situ sensor networks usually need to collaborate with other mobile sensors to collect missing information [6]. In summary, static sensor networks are primarily suitable for areas with pre-identified risks, and they incur a high deployment cost.

As the development of underwater robots, Autonomous Underwater Vehicles (AUVs) are equipped with their own energy source and utilize an autonomous control scheme. Compared with other underwater monitoring platforms, the AUV has higher autonomy and a larger detection range, which is more suitable for monitoring deep-sea suspended sediment plumes. To minimize the distance between the robot and the target location, various control algorithms are employed. These include bang-bang control, Sliding Mode Control (SMC), and Model Predictive Control (MPC), etc. Bang-bang control, known as an algorithm that can suddenly switch between two states, is widely used in situations with a clear distinction between inside and outside. Owing to the limitations of bang-bang control in dealing with dynamic boundaries, researchers have combined it with various methods such as the swarm optimization algorithm and the radial basis function neural network to enhance the effectiveness and robustness of tracking [7,8]. Considering the potential chattering trembling effects caused by switching control algorithms with large gain, Dong and Yao proposed a PI-like SMC algorithm to track an isoline and proved its global convergence [9]. It is particularly effective in static scalar fields. Zhang and Pei utilized the Kriging spatial interpolation method to reconstruct concentration distributions and then applied nonlinear MPC to estimate states during the sampling process [10]. This method tracks the target boundary by minimizing the cost function within predefined constraints.

Although the above control algorithms have their own advantages, only focusing on the control objective can easily lead to inaccurate parameter estimation and failure to locate the target position [11]. Therefore, the estimation and control framework combining methods such as filters, machine learning, and state observers has become the mainstream development direction in boundary tracking, providing an effective approach to addressing the complexity and real-time challenges in dynamic boundary environments. Machine learning methods achieve adaptive control by learning the evolution patterns of boundaries, reducing dependence on precise system models, but still have challenges in real-time performance and computational load [12]. Filters are extensively applied to state estimation in dynamic scalar fields, and their integration with formation control and distributed estimation markedly improves the robustness and real-time performance [13,14]. State observers provide real-time feedback for closed-loop control by estimating system states and external disturbances. In the work of Wang et al., sensors, measuring concentrations in a local range area, were utilized to design a Luenberger partial differential equation observer to infer the distributions of spatial concentration fields [15]. Then, a motion control algorithm was designed for tracking and patrolling the boundary by a single robot, which accomplished rigorous convergence analysis. Subsequently, the sensors based on point-by-point concentration measurement were expanded to design a Luenberger observer with distributed parameters, and the theoretical analysis was extended from two-dimensional space to m-dimensional space [16]. By the formation control for tracking robots and perception robots, the boundary tracking of dynamic plumes was achieved. However, it ignored the inherent errors in the plume model and the measurements. Considering the above problem, Wang and Yao proposed a collaborative control algorithm based on the theory of Lyapunov stability for the leader-follow-anchor unmanned surface vehicles [17]. The leader, the anchor, and the follower achieved the tracking of the dynamic plume boundary and simultaneously formed a uniform distribution on the boundary. Compared with the single platform, the advantages of multi-platform are obvious. It can simultaneously collect the characteristic data from different locations, which can significantly improve efficiency, especially in large-scale dispersion scenarios [18,19,20]. In the context of deep-sea environments, the exchange of information among multiple platforms is subject to various constraints, leading to increased equipment costs, communication loads, and computational complexity. Compared with multiple AUVs, using a single AUV substantially reduces both deployment and maintenance costs, ensuring higher economic efficiency. Additionally, due to the influence of ocean currents, the plume diffusion area is uncertain. Compared with deploying sensor networks, a single AUV can plan and adjust its path according to environmental changes, adapting to various ocean conditions and overcoming the spatial coverage limitations of low-density sensor networks. Therefore, to solve the boundary estimation problem in deep-sea mining, a single AUV demonstrates significant advantages.

In this paper, we address the practical scenario of deep-sea tailing discharge at the bottom of the China Ocean Mineral Resources Association (COMRA) contract area and design a boundary tracking algorithm based on a unified framework of estimation and control for a single AUV. This algorithm, developed from a theoretical and practical perspective, has demonstrated its capability to track a dynamic plume boundary at a fixed speed by a single AUV. The primary contributions of this paper are as follows: (1) A plume model has been constructed based on the suspended sediment dispersion in deep-sea mining, simplified from three-dimensional space to two-dimensional space. (2) The concentration boundary exhibits dynamic characteristics that vary over space and time. Compared with a static boundary, it has higher demands on the accuracy and robustness of the tracking algorithm. Therefore, a real-time control algorithm based on an observer and online estimation has been developed. (3) There are several practical limitations in tracking the boundary. For example, we only know the source location of the tailing, and the sensors with point-by-point measurements have inherent noises and errors. To reduce the effects of disturbance and noise, we construct a Luenberger PDE observer to estimate the dynamic boundary and formulate a control law to guide the AUV to the estimated position. Additionally, the parameters of the model are unknown within the range of experience. To fully utilize known information and enhance the estimation performance of environmental parameters, we add a projection correction unit in adaptive parameter estimation. Thus, all the above-mentioned challenges have been considered and solved in the algorithm design.

The rest of this paper is organized as follows: In Section 2, we present the model of the suspended sediment plume, the simplified AUV model, and the definition of the problem. In Section 3, we introduce a control design for dynamic plume boundary tracking by a single AUV, and we provide a proof for the convergence and stability of the algorithm. In Section 4, numerical simulation results are provided to demonstrate the effectiveness of the proposed control algorithm. Lastly, Section 5 provides a brief conclusion and directions for future work.

## 2. Problem Statement

**Figure 1:**
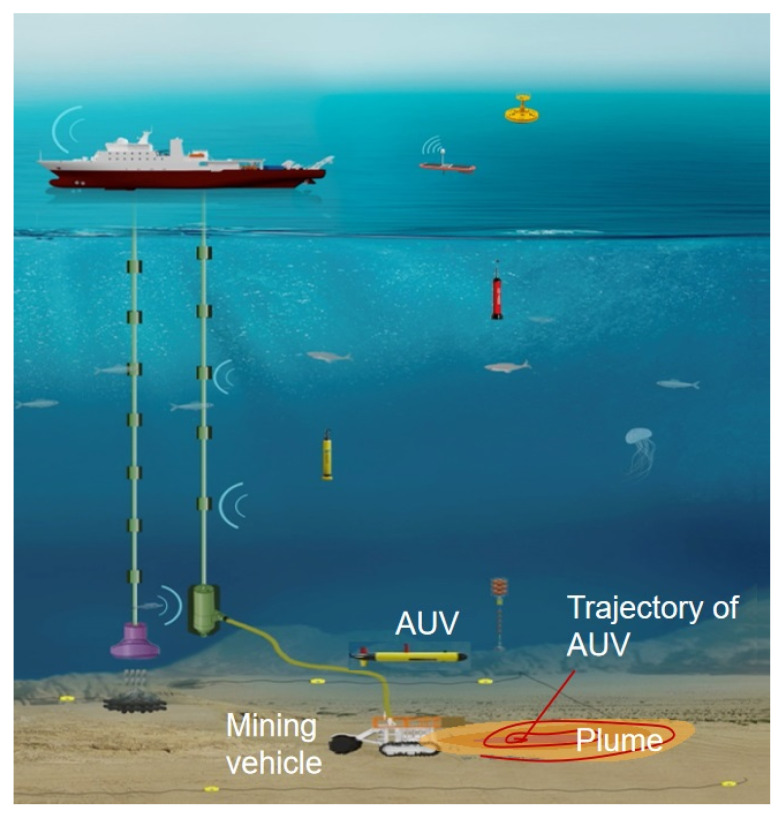
Dynamic plume tracking by single AUV in deep-sea mining.

In this section, we present the suspended sediment plume model and the AUV model and formulate the tracking problem by a single AUV addressed in this paper. The application scenario is shown in Figure 1, where a single AUV monitors the impact range of the deep-sea mining by tracking the dynamic boundary of the plume.

### 2.1. Suspended Sediment Plume Model

The concentration field of suspended sediment plume during deep-sea mining is a scalar dynamic field, where the plume is mainly propagated by the advection effect of ocean currents, the diffusion effect from high concentration to low concentration, and its own settling effect. The behavior of suspended sediment propagation in three dimensions can be illustrated by the following equation [21].

(1) $$\begin{array}{c} \frac{\partial C}{\partial t}+V_{1}\frac{\partial C}{\partial z_{1}}+V_{2}\frac{\partial C}{\partial z_{2}}+V_{3}\frac{\partial C}{\partial z_{3}}+\frac{\partial (w_{s}C)}{\partial z_{3}}=K\nabla^{2}C+q_{s} \end{array}$$

where $z\;=\;[z_{1},z_{2},z_{3}]$ is the position in three-dimensional space, C=C(z,t)≥0 is the sediment concentration of the position *z* at time *t*, $V\;=\;[V_{1},V_{2},V_{3}]$ is the velocity field, $w_{s}$ is the settling velocity, $K\;=\;[K_{1},K_{2},K_{3}]$ is the diffusivity coefficient, $\nabla^{2}C\left(z,t\right)=\frac{\partial^{2}C\left(z,t\right)}{\partial z_{1}}+\frac{\partial^{2}C\left(z,t\right)}{\partial z_{2}}+\frac{\partial^{2}C\left(z,t\right)}{\partial z_{3}}$ is the divergence of *C*, and $q_{s}$ is the source term.

In the process of tailing discharge from the mining vehicle, the larger particles rapidly descend to the seafloor, while the smaller particles stay in the water and move with the ocean currents for a longer time. As the plume of suspended sediment gradually disperses, it remains within a height of 2 m above the sea floor, which is relatively low [22]. Furthermore, the horizontal extent of the marine region is considerably greater than its vertical extent, and the shift in plume boundary along the vertical axis is significantly less than that along the horizontal axis [23]. To facilitate online calculation and real-time tracking, a relatively simple model is essential. Consequently, we can only focus on the propagation of suspended sediment in a two-dimensional area to reduce the computational complexity. The kinematic law for the two-dimensional plume can be expressed in terms of the following advection–diffusion Partial Differential Equation (PDE).

(2) $$\begin{array}{c} \frac{\partial c(x,t)}{\partial t}+v^{T}\left(x,t\right)\nabla c\left(x,t\right)=k\left(t\right)\nabla^{2}c\left(x,t\right),\;t>t_{0} \end{array}$$

Subject to the Dirichlet boundary condition,

(3) $$\begin{array}{c} c\left(x,t\right){|}_{x\in \partial \Omega }=0,t\geq t_{0} \end{array}$$

and the initial condition.

(4) $$\begin{array}{c} c\left(x,t_{0}\right)=c_{0}\left(x\right),x\in \Omega \end{array}$$

where $\Omega \subset R^{2}$ is the given appropriate region, such as a rectangular domain, ∂Ω is the boundary of the region Ω, $x={\left[x_{1},x_{2}\right]}^{T}\in \Omega$ is the position in two-dimensional space, c(x,t)≥0 is the concentration in position *x* at time *t*, $\nabla c\left(x,t\right)=\frac{\partial c(x,t)}{\partial x}$ is the spatial gradient of c(x,t), $\nabla^{2}c\left(x,t\right)=\frac{\partial^{2}c\left(x,t\right)}{\partial x_{1}}+\frac{\partial^{2}c\left(x,t\right)}{\partial x_{2}}$ is the divergence of c(x,t), $\left\{v\left(x,t\right),v\in R^{2}\right\}$ is the advection velocity caused by ocean currents, and {k(t)>0,k∈R} is the diffusivity coefficient.

### 2.2. AUV Model

**Figure 2:**
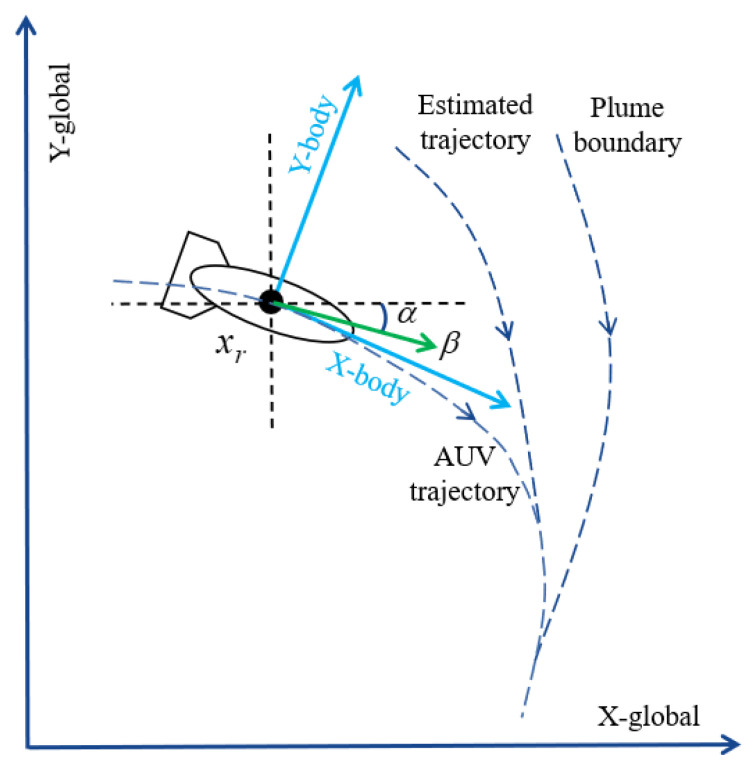
AUV kinematic system in 2D space.

To track the boundary of the two-dimensional plume, we establish the following two-dimensional kinematic model of the AUV [24] as shown in Figure 2.

(5) $$\begin{array}{c} {\dot{x}}_{r}=\left[\begin{array}{cc} \cos\alpha & -\sin\alpha \\ \sin\alpha & \cos\alpha \end{array}\right]\beta \end{array}$$

where $x_{r}={\left[x_{r1},x_{r2}\right]}^{T}$ represents the location of the AUV in the global coordinate system, $\beta ={\left[\beta_{1},\beta_{2}\right]}^{T}$ represents the surge and sway velocities in the body-fixed coordinate system, and α is the heading angle of the AUV.

Since we are predominantly concerned with the AUV’s position, we can define the control input as $u={\left[u_{1},u_{2}\right]}^{T}$, which satisfies the following equation.

(6) $$\beta =\left[\begin{array}{cc} \cos\alpha & \sin\alpha \\ -\sin\alpha & \cos\alpha \end{array}\right]u$$

Thus, a single integrator controller is designed as

(7) $${\dot{x}}_{r}=u.$$

Assuming that the AUV is equipped with the suspended sediment concentration sensor and the positioning unit, we can obtain the concentration value in real time and the accurate location of the AUV. According to the first-order Taylor expansion, the measurement equation for the concentration at position $x_{r}$ can be obtained as follows:

(8) $$y\left(x\right)=\nabla^{T}c_{r}\left(x-x_{r}\right)+c_{r}+w\left(t\right)$$

where y(x)=c(x,t) represents the measurement at time *t*, $x_{r}$ is the position of AUV at time *t*, $c_{r}=c\left(x_{r},t\right)$ is the concentration on position $x_{r}$, $\nabla c_{r}={\left.\frac{\partial c(x,t)}{\partial x}\right|}_{x=x_{r}}$ is the gradient at position $x_{r}$, w(t) is the inherent noise of the sensor. Noting that when the AUV is at the plume boundary position *x*, the measurement concentration satisfies $y\left(x\right)=c_{0}$, where $c_{0}$ is a positive constant.

### 2.3. Problem Formulation

Suppose that if the concentration of suspended particles exceeds the threshold value, it will have a significant impact on the environment. To achieve the objective of monitoring the environmental impact in deep-sea mining, the control objectives can be described as follows:

(1) Assuming the concentration at the plume boundary x(t) is $c_{0}$, the control goal is to design a control law that can drive the AUV tracking the dynamic plume boundary of $c_{0}$.

(2) To achieve smooth monitoring and simplify control design, we set the AUV to a fixed cruising speed $v_{d}$.

Considering the practical conditions of deep-sea mining applications, Assumption 1 is made. Afterwards, the control problem can be defined.

**Assumption A1.**  AUV lacks the Acoustic Doppler Current Profiler, which cannot provide an accurate ocean current flow velocity. Therefore, the advection speed is not measurable, and its true value is defined as $v^{*}$. Furthermore, the diffusion coefficient is isotropic but unknown, and its true value is defined as $k^{*}$. Based on historical data and experience, the range of v and k can be obtained. The information, including the location, the concentration gradient, and the divergence at the location $x_{r}$, can be known.

**Problem 1.**  ***Single AUV Plume Dynamic Boundary Tracking:*** *For the dynamic plume modeled by the advection–diffusion PDE (2-4), we develop a control algorithm for the single AUV with the concentration measurement $\left(y_{r}\left(t\right),t\right)$ to reach the dynamic boundary position $\left\{x\left(t\right)\in R^{2},c\left(x\left(t\right),t\right)=c_{0}\right\}$, and patrol along the boundary with a pre-specified speed $v_{d}\left(t\right)\in R^{2}$, which satisfies Assumption 1.*

## 3. Control Design

In this section, we design a control algorithm based on the framework of observer and online estimation, solving the problem of tracking a dynamic plume in an unknown environment for a single AUV. We construct a Luenberger PDE observer to estimate the expected position of the dynamic boundary, and then we design a control law to drive the AUV to reach the estimated position. On this basis, considering the unknown advection speed and diffusion coefficient, a parameter estimation unit is used to update the parameters. The convergence of the algorithm is analyzed and proven.

### 3.1. Observer Design

As the AUV is expected to patrol along the boundary at a speed of $v_{d}$, the reference point is subject to the following constraint.

(9) $$\frac{{\left(A\nabla c\right)}^{T}}{{∥\nabla c∥}^{2}}\dot{x}=v_{d}$$

where $A=\left[\begin{array}{cc} 0 & -1\\ 1 & 0 \end{array}\right]$ is an orthogonal matrix, *A* is the tangent vector in the direction of plume boundary expansion.

Combined the suspended sediment plume model (2) and the model of AUV (7), the state equation can be derived as follows:

(10) $$\dot{x}=-\frac{(v^{T}\nabla c+k\nabla^{2}c)\nabla c}{{∥\nabla c∥}^{2}}+\frac{v_{d}A\nabla c}{{∥\nabla c∥}^{2}}$$

Regarding the above state Equation (10) and the measurement Equation (8), a Luenberger-type PDE observer can be constructed in the following expression [25].

(11) $$\dot{\hat{x}}=-\frac{\left(v^{T}\nabla c_{\hat{x}}+k\nabla^{2}c_{\hat{x}}\right)\nabla c_{\hat{x}}}{{∥\nabla c_{\hat{x}}∥}^{2}}+\frac{v_{d}A\nabla c_{\hat{x}}}{{∥\nabla c_{\hat{x}}∥}^{2}}-L_{1}\nabla c_{r}\left(\nabla^{T}c_{r}\left(\hat{x}-x_{r}\right)+c_{r}+w\left(t\right)-c_{0}\right)$$

where $\nabla c_{\hat{x}}=\nabla c\left(\hat{x}\right)$ and $\nabla^{2}c_{\hat{x}}=\nabla^{2}c\left(\hat{x}\right)$ are the gradient and divergence at the estimated point $\hat{x}$, respectively, and $g_{1}>0$ is a constant coefficient.

Unfortunately, it is not possible to directly measure or indirectly calculate the values at the estimated point location. The relevant information can only be obtained at the position point $x_{r}$. For remedy, we redefine the observer equation.

(12) $$\dot{\hat{x}}=-\frac{\left(v^{T}\nabla c_{r}+k\nabla^{2}c_{r}\right)\nabla c_{r}}{{∥\nabla c_{r}∥}^{2}}+\frac{v_{d}A\nabla c_{r}}{{∥\nabla c_{r}∥}^{2}}-L_{1}\nabla c_{r}\left(\nabla^{T}c_{r}\left(\hat{x}-x_{r}\right)+c_{r}+w\left(t\right)-c_{0}\right)$$

where $\nabla c_{r}=\nabla c\left(x_{r}\right)$ and $\nabla^{2}c_{r}=\nabla^{2}c\left(x_{r}\right)$ are the gradient and divergence at the AUV location $x_{r}$. However, during the AUV tracking of the estimated point, there exists a control error $e^{\prime }=\hat{x}-x_{r}$. As a result, the replacement of variable information leads to the inherent error in the observer (12). Therefore, we consider eliminating the control error by designing a control law. If $x_{r}$ tends to $\hat{x}$ as time goes to infinity, it means that Equation (12) is equivalent to Equation (11).

### 3.2. Estimation-Based Tracking Control

For the AUV model (7), we add a negative feedback component to reduce the control error $e^{\prime }$. Consequently, we formulate the following control law.

(13) $$\begin{array}{cc} u= & -\frac{\left(v^{T}\nabla c_{r}+k\nabla^{2}c_{r}\right)\nabla c_{r}}{{∥\nabla c_{r}∥}^{2}}+\frac{v_{d}A\nabla c_{r}}{{∥\nabla c_{r}∥}^{2}}\\ \; & -L_{1}\nabla c_{r}\left(\nabla^{T}c_{r}\left(\hat{x}-x_{r}\right)+c_{r}+w\left(t\right)-c_{0}\right)-L_{2}\left(x_{r}-\hat{x}\right) \end{array}$$

where $L_{2}>0$ is a constant coefficient and the term $-L_{2}\left(x_{r}-\hat{x}\right)$ is the negative feedback.

Regarding the observer (12) and the control law (13), the AUV model (7) can be rewritten as ${\dot{x}}_{r}=-L_{2}\left(x_{r}-\hat{x}\right)+\dot{\hat{x}}.$ Thus, we can obtain ${\dot{e}}^{\prime }=-L_{2}e^{\prime }$, which satisfies $\hat{x}\to x_{r}$ as t→∞. However, the parameters *v* and *k* in Formula (13) are unknown, and we only have prior knowledge of their range. Additionally, there exists a noise disturbance w(t). To solve this problem, we design a parameter estimation unit to estimate and update *v* and *k* in the controller. The parameter estimation algorithm can be described as follows [26]:

(14) $$\dot{\hat{c}}={\hat{v}}^{T}\nabla c_{r}+\hat{k}\nabla^{2}c_{r}-\alpha_{1}\left(\hat{c}-c_{r}\right)$$

(15) $$\dot{\hat{v}}=-\alpha_{2}\left(\hat{c}-c_{r}\right)\nabla c_{r}$$

(16) $$\dot{\hat{k}}=-\alpha_{3}\left(\hat{c}-c_{r}\right)\nabla^{2}c_{r}$$

where $\alpha_{1}$, $\alpha_{2}$, and $\alpha_{3}$ are the constant coefficients to adjust the gain of the update, $\hat{v}$ and $\hat{k}$ are the estimated values of *v* and *k*, $\dot{\hat{v}}$, $\dot{\hat{k}}$, and $\dot{\hat{c}}$ are the derivatives of the variables $\hat{v}$, $\hat{k}$, and $\hat{c}$.

Based on Assumption 1, although we cannot obtain the exact value, the ranges of the estimated parameters are known. By constraining the estimated values within the known range, it can accelerate the convergence speed of the parameter estimation, thus improving the control performance of the system. Therefore, we design the projection modification unit to estimate the parameters, which can reduce the error between the estimated value and the true value and drag the system towards a desired set. We define the projection operators of *v* and *k* as follows [27]:

(17) $$Proj_{\Omega v}\left(v\right)=\arg\min_{y\in \Omega v}\left\{\frac{1}{2}{∥v-y∥}^{2}\right\}$$

(18) $$Proj_{\Omega k}\left(k\right)=\arg\min_{y\in \Omega k}\left\{\frac{1}{2}{∥k-y∥}^{2}\right\}$$

where $Proj_{\Omega v}\left(v\right)$ and $Proj_{\Omega k}\left(k\right)$ represent the projection regions of parameters *v* and *k*, {Ωv|v∈Ωv} and {Ωk|k∈Ωk} present the range of unknown parameter *k*.

**Lemma 1**  (See the work of Gaudio et al. [28])**.** *If $\Omega \subset R^{l}$ is a closed convex set, then ${\left(\eta -P_{\Omega }\left(\eta \right)\right)}^{T}\left(P_{\Omega }\left(\eta \right)-y\right)\geq 0,\eta \in R^{l},y\in \Omega$.*

Recalling Formulas (17) and (18), we propose the parameter update laws with projection modification as follows:

(19) $$\dot{\hat{c}}={\hat{v}}^{T}\nabla c_{r}+\hat{k}\nabla^{2}c_{r}-\alpha_{1}\left(\hat{c}-c_{r}\right)$$

(20) $$\dot{\hat{v}}=-\alpha_{2}\left(\hat{c}-c_{r}\right)\nabla c_{r}-\beta_{1}\left(\hat{v}-Proj_{\Omega v}\left(v\right)\right)$$

(21) $$\dot{\hat{k}}=-\alpha_{3}\left(\hat{c}-c_{r}\right)\nabla^{2}c_{r}-\beta_{2}\left(\hat{k}-Proj_{\Omega k}\left(k\right)\right)$$

where $\beta_{1}$ and $\beta_{2}$ are positive constants to adjust the effect of projection operations on the results.

**Figure 3:**
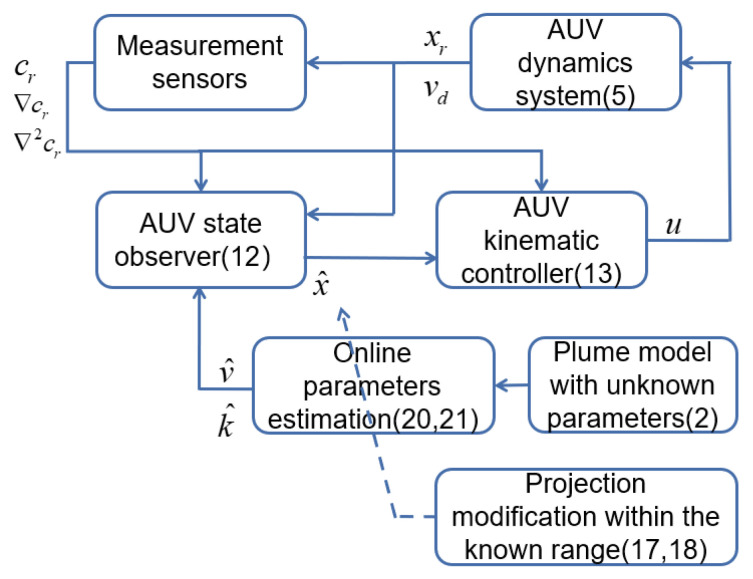
The structure diagram for the single AUV to track the plume boundary.

The structure diagram in Figure 3 illustrates the structure of the single AUV for tracking the plume boundary. Initially, the observer is responsible for estimating the state of the AUV, while online estimation utilizing the projection modification unit solves the unknown parameters in the plume model. Secondly, we obtain the estimated position of the plume boundary to control the AUV. Moreover, the sensors measure the concentration information that is to be used in the observer and controller.

### 3.3. Stability Analysis of System

In this section, we demonstrate the stability of the control system as previously stated. The parameter estimation unit is a crucial part of the scheme, used to estimate the unknown parameters in the unknown suspended sediment plume model. Hence, we use Lyapunov’s stability-based methods to validate its stability at first.

**Theorem 1.**  *If the bounded parameters v and k belong to the convex set, and the coefficients of the parameter estimation unit are positive values, then the parameter estimation system (19)–(21) is globally asymptotically stable.*

**Proof of Theorem 1.**  To simplify the demonstration, we redefine the parameters as $\hat{\eta }={\left[\hat{v},\hat{k}\right]}^{T},\;\alpha =\left[\begin{array}{cc} \alpha_{2} & 0\\ 0 & \alpha_{3} \end{array}\right],$ $\beta =\left[\begin{array}{cc} \beta_{1} & 0\\ 0 & \beta_{2} \end{array}\right],\;h=\left[\begin{array}{cc} \nabla c_{r} & 0\\ 0 & \nabla^{2}c_{r} \end{array}\right]$. By subtracting the Equation (2) from Equation (19), we can obtain Equation (22):

(22) $$\dot{\hat{c}}={\left(\hat{v}-v^{*}\right)}^{T}\nabla c_{r}+\left(\hat{k}-k^{*}\right)\nabla^{2}c_{r}-\alpha_{1}\left(\hat{c}-c^{*}\right)$$

Similarly, we can also derive Equations (23) and (24).

(23) $$\dot{\hat{v}}=-\alpha_{2}\left(\hat{c}-c^{*}\right)\nabla c_{r}-\beta_{1}\left(\hat{v}-Proj_{\Omega v}\left(v\right)\right)$$

(24) $$\dot{\hat{k}}=-\alpha_{*}\left(\hat{c}-c^{*}\right)\nabla^{2}c_{r}-\beta_{2}\left(\hat{k}-Proj_{\Omega k}\left(k\right)\right)$$

Define $\eta^{\prime }=\hat{\eta }-\eta^{*}$, $c^{\prime }=\hat{c}-c^{*}$, and $Proj_{\Omega }\left(\hat{\eta }\right)={\left[Proj_{\Omega v}\left(v\right),Proj_{\Omega k}\left(k\right)\right]}^{T}$, then the estimation unit can be reformulated.

(25) $${\dot{c}}^{\prime }={\eta^{\prime }}^{T}h+\alpha_{1}c^{\prime }$$

(26) $${\dot{\eta }}^{\prime }=-\alpha c^{\prime }h-\beta \left(\hat{\eta }-Proj_{\Omega }\left(\hat{\eta }\right)\right)$$

We formulate the subsequent Lyapunov function candidate for the parameter estimation error system.

(27) $$V=\frac{1}{2}\alpha {∥c^{\prime }∥}^{2}+\frac{1}{2}{∥\eta^{\prime }∥}^{2}$$

The time derivative of the above Lyapunov function can be represented as follows:

(28) $$\begin{array}{cc} \dot{V} & ={\alpha c^{\prime }}^{T}{\dot{c}}^{\prime }+{\eta^{\prime }}^{T}{\dot{\eta }}^{\prime }\\ \; & ={\alpha c^{\prime }}^{T}{\eta^{\prime }}^{T}h-\alpha \alpha_{1}{∥c^{\prime }∥}^{2}-{\alpha \eta^{\prime }}^{T}{c^{\prime }h-\beta \eta^{\prime }}^{T}\left(\hat{\eta }-Proj_{\Omega }\left(\hat{\eta }\right)\right)\\ \; & =-\alpha \alpha_{1}∥c^{\prime }{∥}^{2}-\beta {\eta^{\prime }}^{T}\left(\hat{\eta }-Proj_{\Omega }\left(\hat{\eta }\right)\right) \end{array}$$

Due to α≥0 and $\alpha_{1}\geq 0$, it is obvious that the section $-\alpha \alpha_{1}{∥c^{\prime }∥}^{2}\leq 0$.

By Lemma 1, we can infer $-\beta {\eta^{\prime }}^{T}\left(\hat{\eta }-Proj_{\Omega }\left(\hat{\eta }\right)\right)=-\beta {\left(\hat{\eta }-\eta^{*}\right)}^{T}\left(\hat{\eta }-Proj_{\Omega }\left(\hat{\eta }\right)\right)\leq 0$.

Thus, we can conclude that $\dot{V}\leq 0$, which implies that the parameter estimation error system is globally asymptotically stable. And it can be easily obtained that $\lim_{t\to +\infty }\hat{v}\to v^{*}$ and $\lim_{t\to +\infty }\hat{k}\to k^{*}$. □

Based on the above analysis of the parameter estimation system, the parameter estimation unit can converge the parameters to the true values of the reference model (2). The observer system is exponentially stable; it is that ${∥y\left(x_{r}\right)-\hat{c}∥}^{2}\to 0$ as t→0 [29]. For the purpose of stability analysis of the control system, we defined the following Lyapunov function candidate as

(29) $$V_{2}=\frac{1}{2}{∥e^{\prime }∥}_{2}^{2}$$

The time derivative of $V_{2}$ is given as

(30) $${\dot{V}}_{2}=e^{\prime }{\dot{e}}^{\prime }=-L_{2}e^{\prime 2}=-2L_{2}V_{2}\leq 0.$$

Thus, we can imply that the control system is globally asymptotically stable, which means $\lim_{t\to +\infty }e^{\prime }=0$. It also can be derived $\left|\right|\hat{c}-c_{0}{\left|\right|}^{2}\to 0$ as t→+∞.

Furthermore, by definition the error of the closed-loop system as $e=y\left(x_{r}\right)-c_{0}$, we can derive

(31) $$\begin{array}{cc} {∥e∥}^{2} & ={∥y\left(x_{r}\right)-c_{0}∥}^{2}\\ \; & ={∥\left(y\left(x_{r}\right)-\hat{c}\right)+\left(\hat{c}-c_{0}\right)∥}^{2}\\ & \leq 2{∥y\left(x_{r}\right)-\hat{c}∥}^{2}+2{∥\hat{c}-c_{0}∥}^{2}. \end{array}$$

Hence, we know ${∥e∥}^{2}\to 0$ as t→+∞ (i.e., $c_{r}\to c_{0}$ as t→+∞), which implies that the AUV can track and patrol on the dynamic boundary of the plume.

## 4. Simulations

In this section, we demonstrate the results of tracking the dynamic suspended sediment plume boundary by a single AUV equipped with the point-by-point concentration measurement sensor. The mining tailing was discharged from the bottom of the COMRA contract area in January 2018 at a depth of 6 m, with a discharge rate of 63 kg/s and an average ocean flow velocity of approximately 3.5 cm/s [21]. Based on the given scenario, a simulation environment was constructed to gather information on the plume and validate the proposed tracking algorithm. Meanwhile, we examine two scenarios, one where only the diffusion coefficient is unknown and the other where both the diffusion coefficient and the advection velocity are unknown.

### 4.1. Plume Tracking for Scenario I

In two-dimensional space, a rectangular spatial domain Ω is established as Ω = [0, 20] × [0, 30]. The source, identified as $S_{1}$, is located at [10, 5], where the concentration value is 3. The propagation of suspended sediment in the flow field results in a dynamic concentration field, which is produced by solving the PDE system under the zero initial condition and zero boundary condition. The concentration value of the suspended sediment plume boundary is set to $c_{0}=3$.

For Scenario I, assuming that the advection velocity *v* can be measured, the diffusion coefficient *k* has a range of 0 to 3.5 but cannot be measured. At time t=0, the tailing from deep-sea mining is discharged, which causes the suspended sediment plume to start spreading. At t=2, the AUV is positioned near the point source. In this experiment, the initial position of the AUV is chosen as [9, 7], and the fixed patrol speed of the AUV is set to 4.5 m/s. The control law (13) utilizes $L_{1}$ = 10, $L_{2}$ = 10.

**Figure 4:**
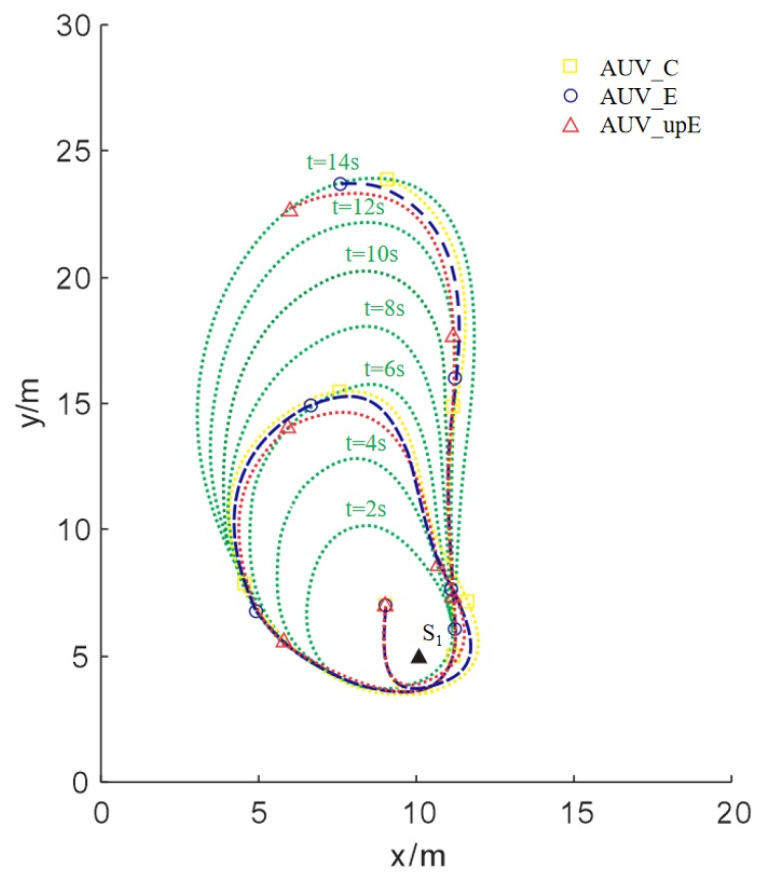
Trajectories of three AUVs with different strategies for Scenario I.

To verify the effectiveness of the algorithm mentioned above, we compare its tracking effects under the following three algorithms. First, we set a fixed diffusion coefficient of *k* = 2.5, referred to as $AUV_{C}$. Subsequently, we set *k* in the parameter estimation process to be updated according to Equations (14) and (16), which is referred to as $AUV_{E}$. Additionally, we integrate a projection modification into the parameter estimation unit according to Equations (17)–(19), which is referred to as $AUV_{upE}$. As time passes, the trajectories of three AUVs are shown in Figure 4. Upon analysis of the results, the following inferences can be drawn. From an overall perspective, when *k* is unknown, all three strategies ultimately converge to the boundary of the plume. However, $AUV_{upE}$ converges to the target boundary in a shorter time with a smaller Mean Squared Error (MSE) between the measured concentration and the boundary concentration.

**Figure 5:**
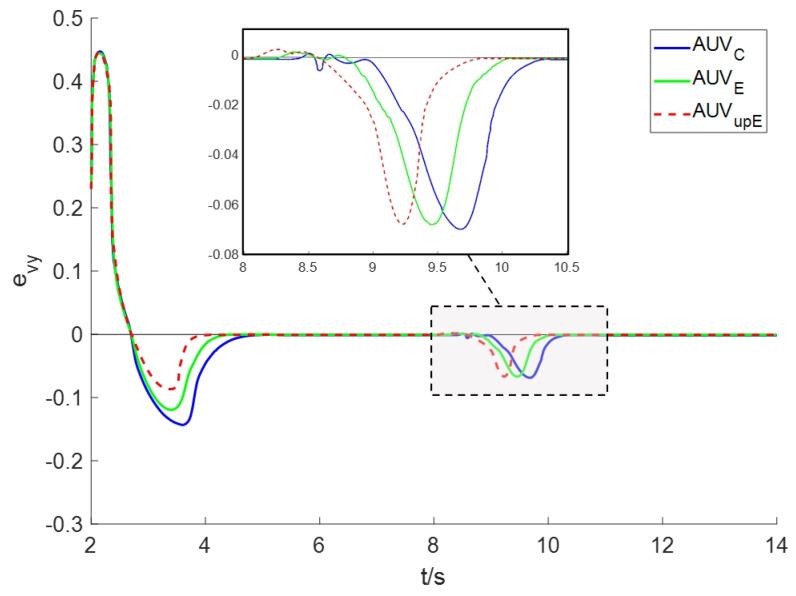
The plume tracking errors for Scenario I.

To further demonstrate the performance of the tracking scheme, Figure 5 displays the plume tracking errors, providing intuitive evidence that $AUV_{upE}$ has superior tracking control effects. It should be noted that when the AUV passes through the lower right corner of the plume area, the concentration error increases. The two peaks occur at approximately *t* = 3.5 s and *t* = 9.5 s in the tracking error. The reason is that this area is close to the point source, where the concentration changes dramatically. Therefore, even minor changes in position can cause significant errors in concentration values.

**Figure 6:**
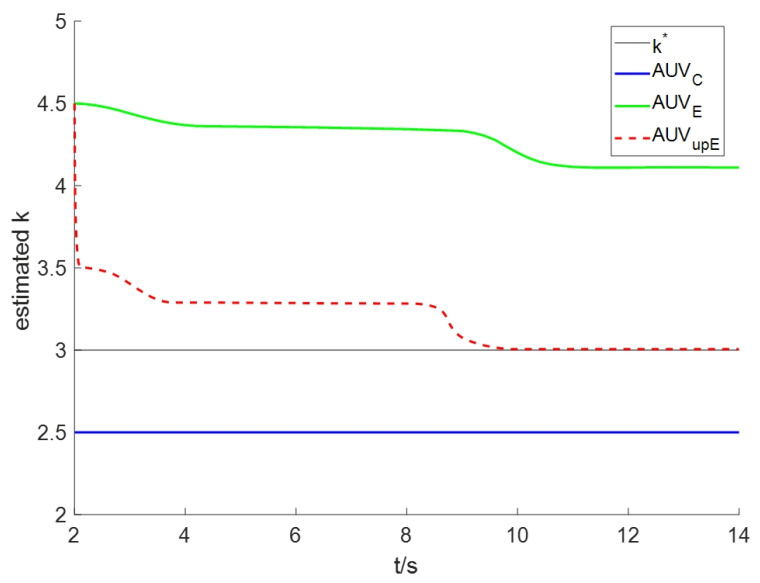
The estimation errors for Scenario I.

To assess the efficacy of online estimation, the estimation errors are shown in Figure 6. It is easily seen that $AUV_{E}$ remains consistently out of the known range of true value, and the estimation system takes a longer time to stabilize. Although the controller can eliminate tracking errors, the estimation performance remains unsatisfactory. Following the initial time, $AUV_{upE}$ quickly enters the known range of true value, and the estimated value eventually converges to the true value. Therefore, the online estimation system with the projection modification unit markedly improves the estimation precision of the diffusion coefficient. Furthermore, there is a fluctuation of the estimated values at around *t* = 9 s, which is also caused by the dramatic change in the measurement concentration.

### 4.2. Plume Tracking for Scenario II

**Figure 7:**
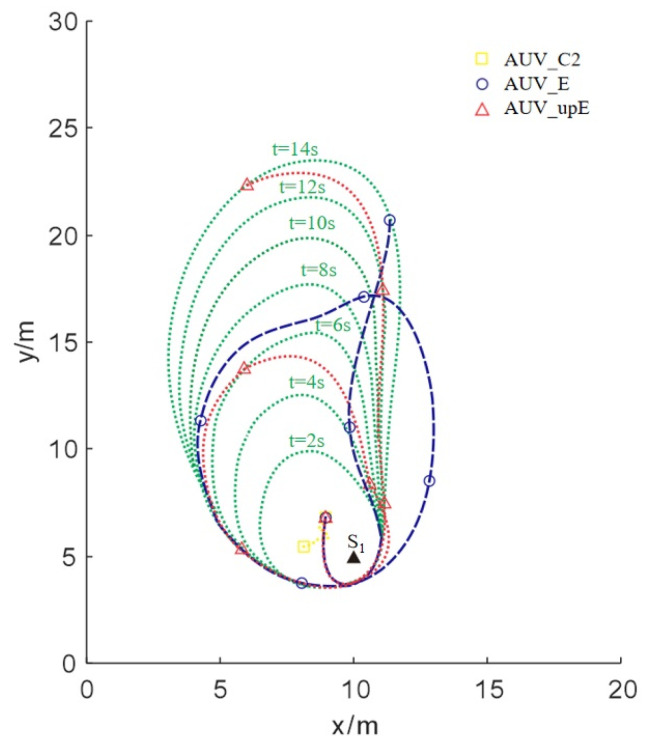
Trajectories of three AUVs with different strategies for Scenario II.

For Scenario II, we assume that both the advection speed *v* and the diffusion coefficient *k* are unknown, with their respective ranges being [−105, 105] and [0, 4]. We compare the tracking effects of the following three algorithms. (1) We set the diffusion coefficient at *k* = 1.75 and the advection speed in the *x* and *y* directions at random values within [−105, 105], which is named $AUV_{C}$. (2) We estimate and update the online parameter according to Equations (14)–(16), which is named $AUV_{E}$. (3) We add projection modification in the estimation unit, updating *v* and *k* according to Equations (17)–(19), which is named $AUV_{upE}$. The simulation results are depicted in Figure 7.

The conclusions are drawn from Figure 7. It is easily seen that $AUV_{C}$ stays near the source point, which cannot track the boundary continuously. $AUV_{E}$ displays significant tracking errors for a prolonged period before eventually converging to the plume boundary position until the end. As time elapses, $AUV_{upE}$ moves around the plume boundary and quickly captures the expansion movement of the plume boundary rapidly. Thus, the rapid convergence and minimal errors demonstrate that the $AUV_{upE}$ exhibits superior tracking performance. Furthermore, compared with Scenario I, the tracking effect of $AUV_{upE}$ is significantly improved compared with that of $AUV_{E}$ for Scenario II.

**Figure 8:**
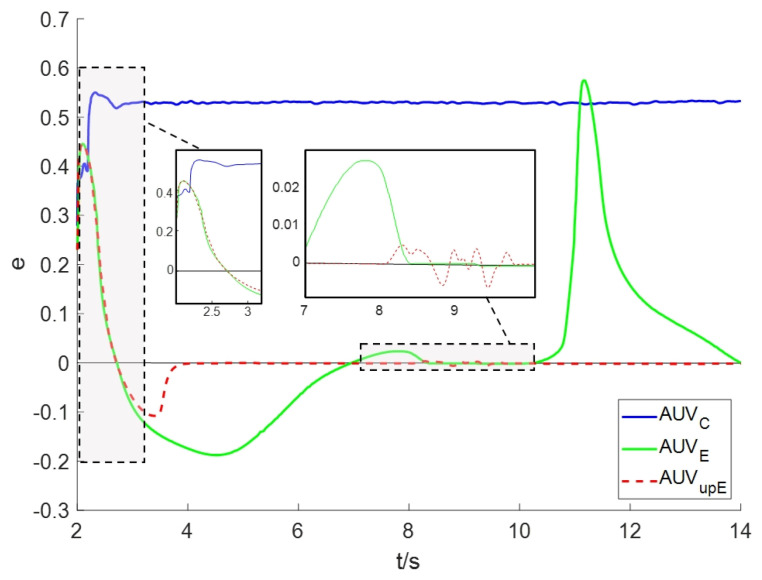
The plume tracking errors for Scenario II.

To further demonstrate the tracking performance of the tracking scheme, we present the tracking errors for Scenario II as shown in Figure 8. Obviously, $AUV_{C}$ cannot eliminate the error after approximately *t* = 3 s. In contrast, $AUV_{E}$ showed significant tracking error at around *t* = 2–8 s and *t* = 10–14 s. However, the tracking error of $AUV_{upE}$ approaches zero rapidly after approximately *t* = 4 s and only exhibits minor fluctuations at *t* = 8–9.5 s. Thus, these results validate the effectiveness of the proposed algorithm in plume tracking for Scenario II.

**Figure 9:**
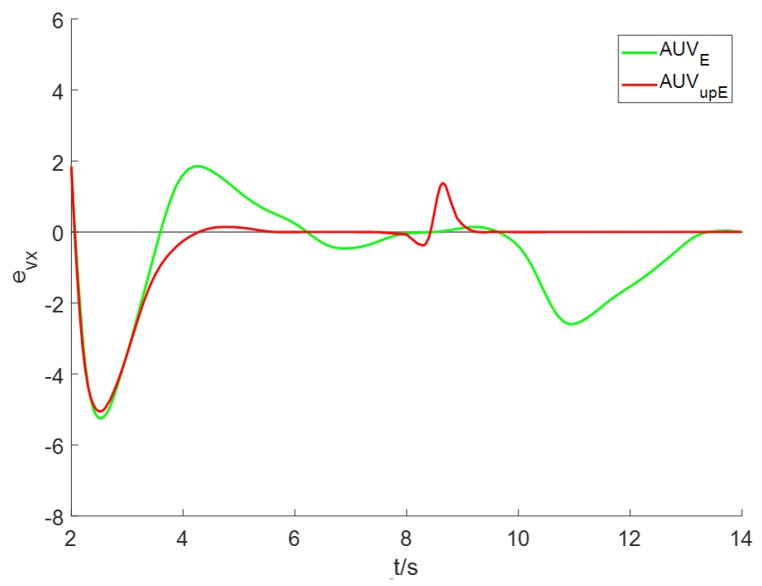
The estimation errors of advection speed in the x-axis directions.

**Figure 10:**
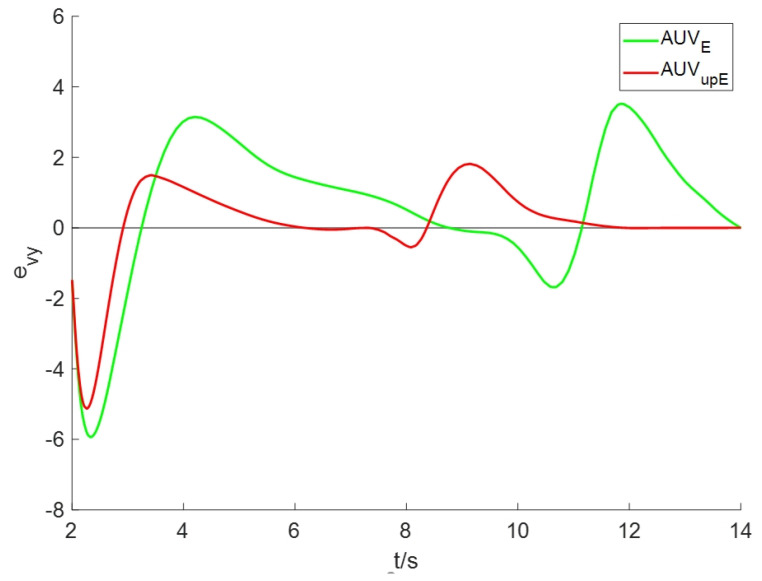
The estimation errors of advection speed in the y-axis directions.

To evaluate the estimation performance of advection speed under the improved algorithm, we define the estimation error of advection speed *v* as $e_{v}=\hat{v}-v^{*}$, where $v^{*}$ is the true value of advection speed at the AUV location in the plume model (2) and $\hat{v}$ is the estimated value of advection speed. Figure 9 and Figure 10 display the estimation errors in the *x*-coordinate and *y*-coordinate directions, respectively. Comparing the estimation error of $AUV_{E}$, it is evident that the improved scheme $AUV_{upE}$ yields a reduced estimation error for *v* in both directions.

## 5. Conclusions

In this paper, the dynamic suspended sediment plume tracking problem is solved by using a single AUV. According to the monitoring conditions, such as point-by-point measurements by sensors and the inherent noise, the tracking scheme is designed based on the structure diagram of estimation and control. To tackle the unknown parameters of the advection–diffusion PDE model, the projection modification is implemented to enhance the performance of the estimation system. By using Lyapunov’s method, we prove the convergence of the estimation system and the stability of the closed-loop system. Numerical simulation results in two cases demonstrate the merits of the proposed tracking scheme. As a key direction for future work, we plan to conduct field trials in real ocean environments to further validate the practical performance of the proposed method. These trials will also take into account several critical real-world challenges, including the influence of seabed topography, the operational performance of AUVs, battery life constraints, and communication limitations. Furthermore, from a practical perspective, it is also essential to extend the 2D advection–diffusion model to the 3D advection–diffusion-settling model and to achieve effective tracking control of plume boundaries in 3D space.

## Data Availability

The data presented in this study are available upon request from the corresponding author.
